# *H. pylori* Infection and Virulence Factors *cagA* and *vacA* (s and m Regions) in Gastric Adenocarcinoma from Pará State, Brazil

**DOI:** 10.3390/pathogens11040414

**Published:** 2022-03-29

**Authors:** Igor Brasil-Costa, Cintya de Oliveira Souza, Leni Célia Reis Monteiro, Maria Elisabete Silva Santos, Edivaldo Herculano Correa De Oliveira, Rommel Mario Rodriguez Burbano

**Affiliations:** 1 Laboratório de Imunologia, Seção de Virologia, Instituto Evandro Chagas, Ananindeua 67030-000, PA, Brazil; 2 Laboratório de Enteroinfecções Bacterianas, Seção de Bacteriologia e Micologia, Instituto Evandro Chagas, Ananindeua 67030-000, PA, Brazil; cintyaoliveira@iec.gov.br (C.d.O.S.); lenimonteiro@iec.gov.br (L.C.R.M.); 3 Instituto de Ciências Biológicas, Universidade Federal do Pará, Belém 66075-110, PA, Brazil; elisabete@ufpa.br; 4 Laboratório de Células e Tecidos, Seção de Meio Ambiente, Instituto Evandro Chagas, Ananindeua 67030-000, PA, Brazil; edivaldooliveira@iec.gov.br; 5 Laboratório de Citogenética Humana, Universidade Federal do Pará, Belém 66075-110, PA, Brazil; rommel@ufpa.br

**Keywords:** *H. pylori*, gastric adenocarcinoma, genetic variability, carcinogenesis, virulence factors

## Abstract

*H. pylori* shows a great variability in genes associated with virulence, which may influence properties related to gastric adenocarcinoma initiation and progression. Among them, *cagA* and *vacA* show a strong positive association with the disease. Therefore, a cross-sectional study was carried out with 281 samples of gastric adenocarcinoma, collected at a cancer reference center in the Brazilian Amazon. Detection of *H. pylori* was proceeded by PCR of the *ureA* and *16S* genes. Positive samples were subjected to the *cagA* detection and *vacA* typing. The bacteria were observed in 32.03% of the samples. Positivity for *H. pylori* was associated with advanced age (*p* = 0.0093) and metastases (*p* = 0.0073). Among the positive cases, 80% (72/90) had the *cagA* gene. For the “s” position of the *vacA* gene, 98.8% (83/84) of the bacteria had genotype s1 and 1.2% (1/84) were genotyped as s2. For the “m” position, the results were: 63.6% (56/88) with m1 genotype, 2.3% (2/88) genotyped as m2 and 34.1% (30/88) m1/m2. Virulence factors did not impact an increase in the association with age or metastases. In conclusion, *H. pylori* infection is associated with malignant phenotype cases of gastric adenocarcinoma, involving metastases. The virulence factors related to the *cagA* and *vacA* genes showed a high prevalence in the Brazilian Amazon.

## 1. Introduction

Since 1994, the World Health Organization (WHO) and the International Agency for Research on Cancer (IARC) determined that there was sufficient evidence to support carcinogenicity of *H. pylori* infection in humans, then classified the bacterium as a class I carcinogen [1]. The infection can cause chronic gastritis, peptic ulcer, gastric adenocarcinoma and lymphoma of the lymphoid tissue associated with the gastric mucosa (MALT) [2,3]. *H. pylori* infection is the main risk factor for stomach cancer [4]. It is a Gram-negative, spiral or slightly curved bacillus, growing in a microaerophilic environment that colonizes the stomach [5]. There is great genomic variability among *H. pylori* strains, which may be associated with genesis or progression of gastric adenocarcinoma, mainly due to the presence of virulence factors associated with the bacterium genome [6,7]. Half of the differentiated genes in each strain are grouped in a hypervariable region, known as the “plasticity zone”. In addition to this “plasticity zone”, the cag pathogenicity island (CPI), a region of approximately 40 Kb that confers virulence, also shows high genetic variability among strains [8,9,10].

Within the CPI, the most studied gene is *cagA*, which differs between strains of *H. pylori* and confers virulence characteristics [9,11,12,13]. This gene is present in about 60% of the strains described in western countries. Positive *cagA* is one of the most virulent strains of *H. pylori*, being associated with gastric ulcer, duodenal ulcer and gastric cancer [14,15].

In some regions of the world, including Asia and most of Africa, almost all strains of *H. pylori* show intact CPI, while 30% of strains of the bacterium found in Europe and North America do not have this island, being considered *cagA* negative. However, it is possible that *cagA* positive and negative strains co-exist in the same stomach [8].

After entering the cytoplasm of gastric cells, CagA is phosphorylated at EPIYA sites (glutamate/proline/isoleucine/tyrosine/alanine) by the family of kinases Abl and Src [16]. Once phosphorylated, the protein can bind to SHP-2 and deregulate the normal signaling process in cells [17]. This action may involve important processes such as the host’s cellular metabolism, including cell proliferation [18], apoptosis and maintenance of normal cytoskeleton structure, prerequisite for neoplastic transformation [16,17,19]. Additionally, the presence of CagA induces an increased production of interleukin 8 by epithelial cells [20,21]. This interleukin produces an intense local inflammatory response and stimulates the production of free radicals such as reactive oxygen species (ROS) that can lead to damage to the DNA of adjacent cells [22]. The accumulation of DNA damage causes genetic changes in gastric epithelial cells, which in turn can culminate in carcinogenesis [15,23].

**Figure 1:**
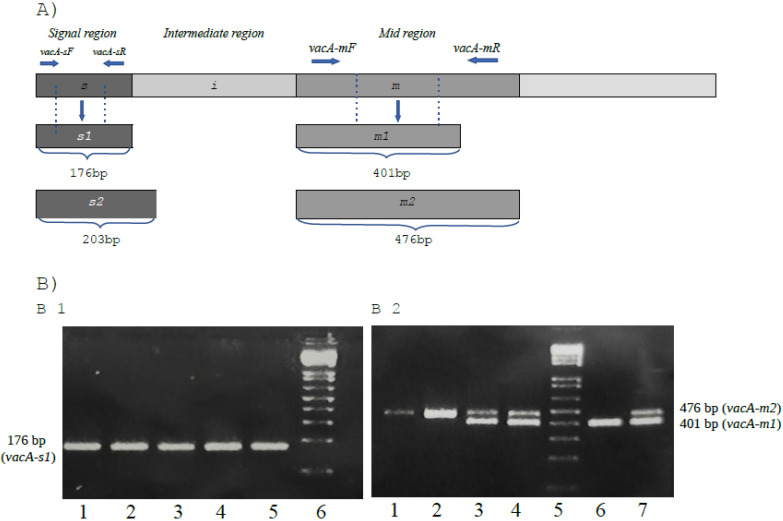
*H. pylori vacA* gene allelic diversity and molecular detection by PCR assay. (**A**) Gene architecture of three regions of the *VacA* gene: the signal region (s1 and s2), the intermediate region (i) and the mid-region (m1 and m2). (**B**) Amplification of *vacA s1* (**B1**) and *vacA m1/m2* (**B2**) alleles. Subtitle: (**B1**) (lane 1 to 4: strains *vacA s1* [176 pb]; lane 5: positive control (*vacA s1*); lane 6: 100 to 1000 pb marker); (**B2**) (lanes 1 and 2: strains *vacA m2* [476 pb]; lanes 3 and 4: strains *vacA m1/m2* [401 pb/476 pb]; lane 5: 100 to 1000 pb marker; lane 6: strains *vacA m1* [401 pb]; lane 7: positive control (*vacA m1/m2*). Note: Result of *vacA s2* not showing in this figure.

In addition to the CPI, *H. pylori* has several other gene regions related to pathogenicity and/or virulence. Among them, we can highlight the genes *vacA*, *iceA* and *babA* [24]. The *vacA* gene is present in the bacterial chromosome and encodes a high molecular weight multimeric protein (VacA) characterized as a multifunctional toxin that induces cell vacuolization, formation of membrane channels, dysregulation of endosomal/lysosomal function, apoptosis and immunomodulation [11,25,26,27,28]. Unlike *cagA*, the *vacA* gene is present in all strains of *H. pylori* but exhibits a high level of genetic diversity. The gene contains two polymorphic regions: the “s” region and the “m” region. The “s” region encodes the signal peptide in the 5′ gene region and can have the s1 or s2 genotypes. In turn, the “m” region is located in the middle region of the gene and encodes the host cell binding site and exists in the m1 or m2 genotypes (Figure 1A). Clinical studies have shown that the s1m1 genotype is more virulent because it has greater vacuolating activity in several cell lines [6,9,12,29,30]. This genotype is associated with an increased production of the vacuolating toxin, culminating in high levels of inflammation and the presence of epithelial damage in the gastric mucosa, which increases the risk of gastric adenocarcinoma [6]. There is also a strong association between the *vacA* s1 genotype and the presence of the CPI, which enhances the virulence of the strain [29,31]. Thus, CagA and VacA are the most important virulence factors during *H. pylori* infection [12,30,32]. 

The present study aimed to determine the prevalence of *H. pylori* infection in gastric adenocarcinomas, as well as the *cagA* gene and s1, s2, m1 and m2 genotypes of the *vacA* gene, associating these factors to gender, age, histological types, primary location tumor and gastric adenocarcinoma staging indices.

## 2. Results

*H. pylori* was detected in 32.03% (90/281) of the samples. The Chi square test demonstrated a borderline statistical association (*p* < 0.05) between the *H. pylori* and clinical-epidemiological information in females, which presented a relatively higher number of positive cases, compared to the males. A significant difference was observed in the analysis of the patients’ age (0.0093). Kruskal–Wallis test showed that the association with age was reinforced (*p* = 0.0005), because positive *H. pylori* patients were older (average of 65.54) compared to negative patients (average of 58.66) (Table 1). 

A robust association was also obtained between positivity for *H. pylori* and the presence of metastases (*p* = 0.0073). Among *H. pylori* positive samples, 59.6% (53/89) had metastases, while only 41.5% (78/188) of the negative ones evolved to this complication (Table 1). The simple logistic regression test reinforced the association of the bacterium with the appearance of metastases (*p* = 0.0053; OR = 2.0762; 95% CI = 1.24 to 3.47).

Among the *H. pylori* positive samples, 80% (72/90) were positive for the *cagA* gene. For the “s” position of the *vacA* gene, 98.8% (83/84) of the bacteria had genotype s1 and 1.2% (1/84) were genotyped as s2, with 6 not obtaining results for the region. For the “m” position, the results were: 63.6% (56/88) with m1 genotype; 2.3% (2/88) genotyped as m2 and 34.1% (30/88) m1/m2, with 2 cases without information from the “m” region (Figure 1B).

For the bacteria combined *vacA* genotype, 63.9% (53/83) were defined as s1m1, 1.2% (1/83) s1m2, 33.7% (28/83) s1m1/m2 and 1.2% (1/83) s2m2, and in seven samples a combined *vacA* genotype were not defined.

The combined *cagA* and *vacA* genotype showed the following result: 53% (44/83) with combined *cagA*+ s1m1 genotype, 30.1% (25/83) with *cagA*+ s1m1/2, 10.9% (9/83) with *cagA*- s1m1, 3.6% (3/83) with *cagA*- s1m1/2, 1.2% (1/83) *cagA*- s1m2 cases and 1.2% (1/83) *cagA*- s2m2.

The comparison of the *H. pylori* genotypes for *cagA*, “s” or “m” *vacA* gene with clinical-epidemiological information demonstrated the association only between patients age and the presence of *cagA* gene (*p* = 0, 0402, by the Chi-square; *p* = 0.0001, by the Kruskal–Wallis test). However, it did not follow the same association tendency with the presence of *H. pylori*, as older individuals were found in the negative *cagA* group (mean of 75.11) when compared with patients diagnosed with the presence of the gene (mean of 61.90).

## 3. Discussion

Although chronic *H. pylori* infection is estimated to contribute to more than 80% of gastric cancer cases [33,34], its prevalence in gastric adenocarcinoma samples varies greatly. For instance, in Asia, where previous studies have consistently reported superior gastric cancer outcomes [35], the prevalence of *H. pylori* in gastric adenocarcinoma samples varies from 40% in China [36] to 89.9% in South Korea [37]. In Brazil, the presence of *H. pylori* showed high prevalence in different regions—85.7% in São Paulo [38,39], 93% in Ceará [9] and 88% in Pará [40]. Despite this, only 32.03% of our samples were positive for *H. pylori* presence, suggesting that other factors may have favored the emergence of the neoplasia [41,42,43,44] in this population, such as genetic constitution, eating habits and other possible exposures. Although *H. pylori* infection is an important promoter step of gastric carcinogenesis, treatment benefits and eradication on gastric cancer prevention is controversial due to intestinal metaplasia and dysplasia be considered a “point of no return” in the precancerous cascade [45,46]. In this sense, cancer occurrence is observed, however, the bacteria detection is negative. In addition, other microbiological and molecular aspect in detection may have influenced the low prevalence found in this study, such as the low-threshold bacterial activity or low-copy *H. pylori* load occurs in the patients with gastric atrophy, intestinal metaplasia, gastric mucosa-associated lymphoid tissue (MALT) lymphoma and upper gastrointestinal bleeding [47].

The presence of *H. pylori* was slightly higher in females (*p* = 0.0437); however, we judge this result as a statistical artifact due to the lack of biological basis for the phenomenon and because the epidemiological studies point to an equal proportion between men and women infected with *H. pylori* or increased risk of infection among the male population [12,42,43]. As gastric cancer belongs to a set of complex diseases, it has a multifactorial character in terms of risk factors, leading to some erroneous statistical findings [44], since hardly all factors that contribute to the outcome will be controlled.

The bacterium is normally associated with the intestinal type of gastric adenocarcinoma, with distal or non-cardiac location and with the presence of distant metastases [40,48,49]. However, a study also associated *H. pylori* with the cardiac location [50]. The same frequency is observed in different degrees of metastasis [51], however, in the present study, the association of the presence of bacteria in metastatic cancer strongly associated only with the presence of metastases (*p* = 0.0073, by X2 and *p* = 0.0053 by RLS). Kong et al. demonstrated that the bacteria can positively regulate the expression of CACUL1, a protein associated with the cell cycle that promotes cell proliferation by activating CDK2 in the transition from G1 to S phase [52]. CACUL1 is also capable to stimulate the expression of the *MMP-9* and *SLUG* genes, important in the process of cell invasion and migration, which confers a great risk of metastases. Infection by the bacterium can produce a more invasive phenotype of malignant cells by releasing gamma-glutamyltransferase, which contributes significantly to the establishment of metastases [26]. A higher expression of *MMP-7* was also found in patients infected with *H. pylori* [53,54]. Bagheri et al. observed an increased expression of MMP9 in non-neoplastic diseases of the stomach in patients infected with bacteria, suggesting that changes occur in premalignant stages [53].

As for the bacterial virulence factors, the *cagA* gene was found in 80% of the strains. A study carried out in the same geographic region, addressing the same neoplasia, observed the presence of the virulence gene in 88.3% of cases [41]. Another study, developed in the northeast region of Brazil, found 64.9% of the strains showing the gene [9]. The association found between the presence of *cagA* and younger patients suggests parallel risks for the development of gastric adenocarcinoma, where these patients develop the neoplasia primarily in the presence of the virulence factor, while in the absence of the factor, the bacterium is favored by senility. Additionally, the presence of other pathogenicity island genes may confer greater or lesser effectiveness on the carcinogenic action of CagA [55]. 

It is important to highlight the high number of cases with mixed infection by more than one strain: 33.7% of the *vacA* genotypes were determined as m1/m2. This result reflects an increased exposure to the transmission of the bacterium or an accumulation of strains due to the failure to resolve the first infection [56]. Another study, in the state of Pará, Brazil, found only 2.4% of infections by more than one strain [41]. It is suggested that there is a greater susceptibility to the persistence of *H. pylori* in the studied population; however, further studies are needed to better elucidate this finding. A study carried out in Colombia demonstrated that there is a greater number of infections with multiple strains and greater bacterial genetic variability in regions of greater risk for the development of gastric cancer, when compared to regions of lower risk [57].

*H. pylori* genotype associated as a risk factor (*cagA*+ s1m1) for gastric adenocarcinoma was found in 83.1% of cases, a percentage similar to that observed by Vinagre et al. at 86.9%, also in the state of Pará, Brazil. This result demonstrates that the strains of bacteria we have found have great oncogenic potential [41], which could explain the high prevalence of gastric adenocarcinoma in the studied population.

The associations found between the presence of the bacterium and the clinical-epidemiological variables had a loss of statistical significance when comparing only the genotypes considered more virulent (*cagA*+, s1, m1, s1m1 and *cagA*+ s1m1). Some studies observed an association with the development of gastric cancer and the presence of metastases for the *cagA*+ strains, unlike our results [40,58,59]. This may be due to the multifactorial nature of the disease. However, Dabiri et al. found no association between the presence of *cagA* and the neoplasm, demonstrating that the presence of this virulence factor is still controversial for the increased risk of developing gastric cancer and more accelerated disease progression [58]. A hypothesis to justify the divergent results may be the genetic variability that is not normally observed in most studies, such as the number of copies of *cagA* (ranging from 1 to 4) and the ignored variability in regions of low coverage by sequencing [60,61]. On the other hand, the involvement of other virulent factors cannot be ignored [24,29,62]. Gastli et al. suggest the use of *H. pylori* quantification associated with *cagA* genotyping in routine workflow for a sensitive and reliable diagnosis, to identify patients at high risk and to manage eradication therapies [62].

Regardless of the strain, the importance of *H. pylori* in the development of gastric cancer appears to be indisputable. The eradication of the bacteria by itself causes a significant decline in cases of the disease [63,64,65,66,67,68,69]. In addition to the eradication of the bacteria with antibiotics, new approaches to gastric cancer prevention have emerged, such as piperine treatment, which suppresses *H. pylori* toxin entry into gastric epithelium and minimizes β-catenin mediated oncogenesis and IL-8 secretion in vitro [70].

## 4. Materials and Methods

A cross-sectional study was carried out with 281 DNA samples extracted from fresh tumor biopsies from cases of gastric adenocarcinoma collected at the Ophir Loyola Hospital, State of Pará, Brazil, a reference in diagnosis and treatment for this tumor type. Histological types were classified as intestinal or diffuse. Epidemiological and histological information was obtained from the Human Cytogenetics Laboratory gastric tumor database, Federal University of Pará (Brazil).

DNA samples were extracted using the QIAamp tissue kit (Qiagen, Hilden, Germany), according to the manufacturer’s protocol, and subjected to quantification with the Qubit^®^ dsDNA BR Assay kit on the Qubit^®^ equipment (Life Technologies, Carlsbad, CA, USA). All samples with concentrations higher or lower than 100 ng/µL were adjusted for this value.

Molecular detection of *H. pylori* was performed by PCR, using two target regions: the urease A (*ureA*) genes and the *16SrRNA* (16S) gene. Positive samples for at least one of these genes were directed to PCR [32] for the *cagA* virulence gene (cytotoxin-associated gene A) and genotyping through the allelic search for the *vacA* gene (vacuolating cytotoxin gene A): signal region (s1/s2) and median region of the gene (m1/m2). The PCR primers to search for these genes and the sizes of the amplified fragments are shown in Table 2.

The PCR reactions were performed with a final volume of 25 µL, using the components and their respective concentrations and volumes according to the specific target as observed in Table 3.

To perform the cycling, the thermal cycler Veriti Thermal Cycler (Applied Biosystems, Waltham, MA, USA) was used. Cycling programs were performed at a denaturation temperature of 95 °C for 5 min, followed by 40 cycles of denaturation, annealing and extension for one minute each and finally an extension step of 7 min at 72 °C. For the diagnostic targets *16SrRNA* and *ureA*, annealing temperatures of 50 °C and 60 °C, respectively, were used. For the virulence factor targets *cagA* and *vacA*, the annealing temperature was 55 °C.

For positive control of the reactions for *H. pylori*, ATCC 43504 strain and DNA extracted from biopsies with positive urease test were used, while DNA extracted from biopsies with negative urease test were used as negative control.

The PCR products were subjected to electrophoresis on 2.0% agarose gel containing 6 µL of Sybr Safe (Invitrogen, Waltham, MA, USA) for visualization and determination of the sizes of the amplified DNA fragments. Together with the samples, the positive controls and the molecular weight marker (Ready-load 1 kb DNA Ladder, Invitrogen) were applied to the gels for comparison. Electrophoretic migrations were performed under constant voltage of 100 V in TE buffer (Tris-EDTA Buffer) for 45 min. After the migration, the gels were visualized on a transilluminator (ultraviolet light) for visual analysis of the amplified DNA fragments, and the photographic record was performed by the UPV Biolmaging Systems (EpiChemi Darkroom, Singapore) photo-documentation system.

Bioestat v5.3 (https://www.mamiraua.org.br/downloads/programas/, accessed on 30 October 2021) was used to perform the statistical analyses. Simple and multiple logistic regression tests, chi-square, and Kruskal–Wallis were conducted to verify associations between the epidemiological and histological variables with the presence or factors of *H. pylori* virulence.

## 5. Conclusions

Our study demonstrated that the prevalence of *H. pylori* infection in gastric adenocarcinoma in the State of Pará, Brazilian Amazon, was considerably lower when compared to other similar studies, however, the majority of positive cases had the usual more virulent strains. The presence of the bacteria was associated with advanced age and the presence of metastases, reinforcing the importance of infection for the progression of the neoplasia. Our results suggest that the bacteria may not be present throughout the course of gastric adenocarcinoma, but it has the potential virulence to accelerate the development of the disease in the Brazilian Amazon.

## Figures and Tables

**Table 1:** Comparison of positivity for *H. pylori* with clinical-epidemiological variables.

Clinical and Epidemiological Variable		*Positive H. pylori*	*Undetectable H. pylori*	Value of *p*
Age	≥60	65.6% (59/90)	48.2% (92/191)	0.0093
	<60	34.4% (31/90)	51.8% (99/191)	
Gender	Man	60% (54/90)	72.8% (139/191)	0.0437
	Woman	40% (36/90)	27.2% (52/191)	
Location	Proximal	37.8% (34/90)	42.4% (81/191)	0.4614
	Distal	62.2% (56/90)	57.6% (110/191)	
Histological type	Intestinal	53.3% (48/90)	57.1% (109/191)	0.5563
	Diffuse	46.7% (42/90)	42.9% (82/191)	
Presence of metastases	presence	59.6% (53/89)	41.5% (78/188)	0.0073
	Absent	40.4% (36/89)	58.5% (110/188)	

**Table 2:** Description of PCR primers used for molecular detection and genotyping of *H. pylori*.

Target	Primer PCR	Size (pb)	Reference
*ureA*	5′-GCCAATGGTAAATTAGTT-3′	394	[64]
	5′-CTCCTTAATTGTTTTTAC-3′		
*16SrRNA*	5′-CCCATTTGACTCAATGCGATG-3′	132	[65]
	5′-TGGGATTAGCGAGTATGTCGG-3′		
*cagA*	5′-GTGCCTGCTAGTTTGTCAGCG-3′	402	[66]
	5′-TTGGAAACCACCTTTTGTATTAGC-3′		
*vacA* m1/m2	5′-CACAGCCACTTTCAATAACGA-3′	401/476	[67]
	5′-CGTCAAAATAATTCCAAGGG-3′		
*vacA* s1/s2	5′-ATGGAAATACAACAAACACAC-3′	176/203	[67]
	5′-CCTGARACCGTTCCTACAGC-3′		

**Table 3:** Components, concentrations and volumes of PCR constituents for molecular detection and genotyping.

Components/Concentrations	Volumes	
	*16SrRNA*	*ureA, cagA, vacA*
Ultra-pure water	14.55 μL	16.55 μL
Reaction buffer 10X (Invitrogen)	2.5 μL	2.5 μL
Magnesium chloride-MgCl_2_ 50 mM (Invitrogen)	0.5 μL	0.5 μL
Deoxynucleotides-dNTP 10 mM (Invitrogen)	1.0 µL	1.0 µL
Oligonucleotides-10 pmol/μL (Invitrogen)	1.0 μL/1.0 μL	1.0 μL/1.0 μL
Platinum-*Taq* DNA Polymerase 5 U/µL (Invitrogen)	0.2 μL	0.2 μL
DNA extracted from gastric biopsy	4 μL	2 μL
Final reaction volume	25 μL	25 μL

## Data Availability

The data presented in this study are available on request from the corresponding author.
